# Thanks to our peer reviewers

**DOI:** 10.1002/lrh2.10349

**Published:** 2022-10-06

The publication of Issue 4 marks the completion of **Volume 6** of *Learning Health Systems*. An open access publication, the journal has advanced research and scholarship on learning health systems in partnership with our reviewers. With indexing in multiple major sources and the recent news that we will have an official impact factor next year, we believe the journal is now on a sustainable, positive trajectory.

We were also delighted to learn that articles from the journal were downloaded over 100 000 times in 2021.

The journal has also published six *Special Issues*: “Patient Empowerment and the Learning Health System” (v.1); “Ethical, Legal, and Social Implications of Learning Health Systems” (v.2); “Learning Health Systems: Connecting Research to Practice Worldwide” (v.3); “Human Phenomics and the Learning Health System” (v.4); “Collaborative Learning Health Systems: Science and Practice” (v.5); and “Education To Meet the Multidisciplinary Workforce Needs of Learning Health Systems” (v.6). Our talented guest editors have been instrumental in helping these *Special Issues* come to fruition.

We are keenly aware that these achievements would not have happened without the dedicated efforts and insightful comments of all those individuals who accepted invitations to review submitted articles. With busy schedules and full commitments in the midst of the Covid‐19 pandemic, these individuals found the time and energy to contribute their expertise to our authors to help ensure that their papers met (and often exceeded) the journal's high standards for publication.

Please accept our sincere gratitude for your outstanding efforts.

*Charles P. Friedman*, Editor in Chief

**REVIEWERS FOR VOLUME 6 LHS JOURNAL**

Includes reviewers of rejected manuscripts

Julia Adler‐Milstein (United States)

Joan Ash (United States)

Mark Ashworth (United Kingdom of Great Britain and Northern Ireland)

Ross Bailie (Australia)

Eta Berner (United States)

Jeff Brown (United States)

Michael Cantor (United States)

Jim Cimino (United States)

Derek Corrigan (Ireland)

Vasa Curcin (United Kingdom of Great Britain and Northern Ireland)

Brendan Delaney (United Kingdom of Great Britain and Northern Ireland)

Catherine Diederich (United States)

Deborah Dinardo (United States)

Gerry Douglas (United States)

Archie Drake (United Kingdom of Great Britain and Northern Ireland)

Christine Dymek (United States)

Margo Edmunds (United States)

Jordan Everson (United States)

Stephan Fihn (United States)

Erin P. Finley (United States)

Allen Flynn (United States)

Thomas Foley (United Kingdom of Great Britain and Northern Ireland)

Emily Ginier (United States)

Shaun Grannis (United States)

Sarah Greene (United States)

Jeanne‐Marie Guise (United States)

W. Ed Hammond (United States)

Kristi Holmes (United States)

Kelli Johnson (United States)

Owen Johnson (United Kingdom of Great Britain and Northern Ireland)

Dipak Kalra (Belgium)

Amy Kilbourne (United States)

Rebecca Kush (United States)

Elyse Lasser (United States)

Harold Lehmann (United States)

Christopher Longhurst (United States)

Nancy Lorenzi (United States)

David McCallie (United States)

Mark McGilchrist (United Kingdom of Great Britain and Northern Ireland)

Marianne McPherson (United States)

Brad Malin (United States)

Kenneth Mandl (United States)

Erik Mayer (United Kingdom of Great Britain and Northern Ireland)

Daniella Meeker (United States)

Paul Meissner (United States)

Michelle Mengeling (United States)

Blackford Middleton (United States)

Tara Montgomery (United States)

David Mosen (United States)

Cheryl Moyer (United States)

Mark Musen (United States)

Paige Nong (United States)

Sally Okun (United States)

Nancy Pandhi (United States)

Gareth Parry (United States)

Niels Peek (United Kingdom of Great Britain and Northern Ireland)

Jodyn Platt (United States)

Rachel Richesson (United States)

Jamie Roberts (United States)

Joshua Rubin (United States)

Philip Scott (United Kingdom of Great Britain and Northern Ireland)

Ruth Simmons (United States)

Karandeep Singh (United States)

Carolynn Smith (Australia)

Jean Soler (Malta)

Caren Stalberg (United States)

Zachary Strasser (United States)

Ndidi Unaka (United States)

Kim Unerti (United States)

Barbara Van de Castle (United States)

Robert Verheij (Netherlands)

Sam Volchenboum (United States)

Jim Walker (United States)

Denise Warzel (United States)

Terrie Wheeler (United States)

Paul Wicks (United States)

